# Drug Resistance to Integrase Strand-Transfer Inhibitors among HIV-1-Infected Adults in Guangdong, China

**DOI:** 10.3390/pathogens11111321

**Published:** 2022-11-10

**Authors:** Yun Lan, Linghua Li, Ruolei Xin, Xuemei Ling, Xizi Deng, Junbin Li, Liya Li, Weiping Cai, Feng Li, Fengyu Hu

**Affiliations:** 1 Guangzhou Eighth People’s Hospital, Guangzhou Medical University, 8 Huaying Road, Baiyun District, Guangzhou 510440, China; 2 Beijing Center for Disease Prevention and Control, Beijing 100013, China; 3 Guangdong Center for Diagnosis and Treatment of AIDS, 627 Dongfeng East Road, Yuexiu District, Guangzhou 510060, China

**Keywords:** HIV-1, drug resistance mutations, integrase strand transfer inhibitors, drug resistance

## Abstract

Background: Integrase strand-transfer inhibitor (INSTI)-containing regimens have gradually been administered in Guangdong Province, China beginning in 2016, and INSTI-related drug resistance (DR) may occur and should be monitored among HIV-1-infected patients. Objective: To investigate the prevalence of INSTI-related resistance among HIV-1-infected individuals in Guangdong and provide evidence for the optimal administration of INSTIs. Methods: This study recruited 1208 HIV-1-infected patients (including 404 ART-naive and 804 ART-experienced patients) between June 2021 and April 2022. The entire integrase gene was amplified from blood plasma. Demographic and epidemiological information were collected. INSTI mutations and susceptibility were interpreted using the Stanford HIV Drug Resistance Database HIVdb program. Results: Of the 1208 enrolled individuals, 2.65% (32/1208) carried at least one INSTI major or accessory drug resistance mutation (DRM), with 1.49% (6/404) being from ART-naive individuals and 3.23% (26/804) from ART-experienced individuals. Among them, seven polymorphic major mutations were detected. Although no INSTI drug resistance was found among treatment-naive patients, seven ART-experienced patients (0.87%, 7/804) carried mutations conferring resistance to INSTIs. Conclusion: The overall prevalence of INSTI DRMs and DR was comparatively low among ART-naive and ART-treated populations in Guangdong; however, INSTI-related polymorphic mutations were observed. Surveillance should be reinforced before transfer to INSTI-containing regimens.

## 1. Introduction

The HIV epidemic remains one of the most serious global health threats [1,2]. The World Health Organization (WHO) has estimated that approximately 37.7 million people were living with HIV infection globally in 2020, with 1.5 million new infections with HIV and 0.68 million HIV-related deaths [3]. About 1.05 million people living with HIV and AIDS (PLWHIV), with another 351 thousand deaths, were cumulatively reported in China by the end of 2020 [4]. Heterosexual contact accounted for 74.2% newly diagnosed HIV infections, while men who have sex with men accounted for 23.3% in 2020. In Guangdong, a cumulative 78.2 thousand PLWHIV were reported by the end of October 2021, with 8.8 thousand individuals newly diagnosed in Guangdong during January to October of 2021 [5].

Combined antiretroviral therapy (cART) suppresses HIV replication, allowing immune reconstitution to occur, dramatically decreasing HIV/AIDS-related morbidity and mortality as well as the risk of further HIV transmission [6,7]. By the end of 2020, approximately 73% (27.5/37.7 million) of PLWHIV globally were receiving ART [3]. By the end of October 2021, 85.67% (67.0/78.2 thousand) of PLWHIV in Guangdong were receiving ART [5].

Overall, increased use of reverse transcriptase inhibitor-based regimens has been accompanied by the emergence of drug resistance, which leads to treatment failure and can be transmitted to those with new infections [8,9,10,11]. Indeed, a multicountry HIV drug resistance analysis in five sub-Saharan African countries showed that 53.0% and 8.8% of newly diagnosed infants exhibited resistance to one or more nonnucleoside reverse transcriptase inhibitors (NNRTIs) or nucleoside reverse transcriptase inhibitors (NRTIs), even before treatment was initiated [12].

Integrase strand-transfer inhibitors (INSTIs) are the latest class of drugs available that target the HIV integrase enzyme; they offer novel treatment options for patients with both ART-naive and acquired or transmitted drug resistance (ADR or TDR) to protease inhibitors (PIs) and NNRTIs [13,14]. The first-generation INSTIs raltegravir (RAL) and elvitegravir (EVG) were approved by the Food and Drug Administration for clinical use in 2007 and 2012, respectively; the second-generation INSTIs dolutegravir (DTG), bictegravir (BIC), and cabotegravir (CAB) were approved in 2012, 2018, and 2021, respectively [15]. In US Department of Health and Human Services Adult and Adolescent HIV treatment guidelines, one INSTI plus two NRTIs are recommended regimens for ART-naive patients [13,16]. RAL or DTG plus two NRTI regimens are recommended as first-line treatment in the 2018 Chinese Guidelines for Diagnosis and Treatment of HIV/AIDS [17], and have been widely used in Guangdong since 2016.

Although INSTIs have been proven to be an effective antiretroviral drug against HIV infection [18,19], the occurrence of drug resistance mutations may be inevitable [20]. In the present study, we conducted a province-wide survey to investigate INSTI resistance mutations and drug susceptibility among ART-naive and ART-experienced patients in Guangdong. Because drug resistance to INSTIs is not routinely included in HIV-1 genotypic testing in China, our results provide important evidence for clinicians as well as for the development of preventive HIV/AIDS control strategies.

## 2. Materials and Methods

### 2.1. Study Population and Data Collection

HIV-1-infected individuals were eligible for inclusion in this study if they were 16 years old or older, treatment-naive, or ART-experienced with HIV-1 viral load above 1000 copies/mL. We recruited 1208 individuals from Guangdong between June 2021 and April 2022, comprising 804 ART-experienced and 404 ART-naive individuals. Epidemiological data for the patients (including age, sex, transmission route, geographical region, year at diagnosis, and CD4+ T-cell counts) were downloaded from the National Free Antiretroviral Treatment Database for Disease Control and Prevention.

### 2.2. Sample Collection, Viral Load Determination, and Genotyping

Anticoagulant EDTA peripheral blood samples were collected and plasma was separated after centrifugation. Real-time molecular beacon detection was applied to detect HIV-1 viral load (Daan, China). HIV-1 genotypes were determined using the online tool COMET HIV (https://comet.lih.lu/index.php, accessed on 8 November 2022) and confirmed using a maximum likelihood (ML) phylogenetic tree based on protease (PR) and partial reverse transcriptase (RT) sequences.

### 2.3. RNA Extraction, Amplification, and Sanger Sequencing

Viral RNA was extracted from plasma samples using an automatic magnetic-bead-based Virus RNA Extraction Kit (Daan, China) according to the manufacturer’s instructions. Amplification of the entire Integrase (IN) gene (HXB2 4230–5093, covering all 288 amino acids of integrase) was performed with an in-house RT–PCR procedure, as previously described [21]. Positive PCR products were separated by agarose gel electrophoresis and sent to Tianyi Huiyuan Genomics Company for Sanger sequencing.

### 2.4. Genotype Identification and Genotypic Drug Resistance Analysis

The obtained sequences were assembled and cleaned with Sequencher DNA sequence analysis software (Version 5.4.6) and then aligned using BioEdit software (Version 7.2). The Stanford HIV-1 drug resistance database (HIVdb version 9.1, https://hivdb.stanford.edu/hivdb/by-sequences/, accessed on 2 June 2022) was employed to identify INSTI mutations and sensitivity to BIC, CAB, DTG, EVG, and RAL. Sequences associated with low-level, intermediate, or high-level categories of resistance were defined as conferring INSTI drug resistance.

### 2.5. Sequence Data and Gene Evolution Analysis

All IN sequences from the 1208 HIV-1 individuals were submitted to the GenBank website (https://www.ncbi.nlm.nih.gov/genbank, accessed on 22 July 2022) under accession numbers OP032752-OP033959.

The ML phylogenetic tree was generated using PhyML version 3.0 (https://www.hiv.lanl.gov/content/sequence/PHYML/interface.html, accessed on 28 September 2017) with the GTR model [22]. Branch support was estimated using the approximate likelihood ratio test (aLRT) SH-like supports, and aLTR values higher than 0.9 were used to identify lineages. The final trees were visualized using Figtree V1.4.2.

### 2.6. Statistical Analysis

All statistical analyses were performed using IBM SPSS V25.0. Quantitative statistics were described using the median (IQR). The chi-square test was applied for comparisons between two groups, and the level of significance for the evaluation of two-sided *p* values was set at 0.05.

## 3. Results

### 3.1. Demographic Information of the Study Participants

In total, 1208 individuals were included in this study, including 404 ART-naive and 804 ART-experienced individuals. The age of the participants ranged from 16 to 87 years, with a median age of 43 years. Among them, 50.99% (616/1208) were recruited in 2021 and 49.01% (592/1208) in 2022. Approximately 44.04% of the individuals had confirmed infections before 2019. Most of the subjects (81.85%, 990/1208) were male. Heterosexual (HET) contact comprised the predominant risk group (59.02%, 713/1208), followed by men who have sex with men (MSM) (26.99%, 326/1208) and intravenous drug users (IDU) (7.37%, 89/1208). The median HIV-1 RNA viral load at the time of the drug resistance test and the CD4+ T-cell count at baseline were 4.15 (log 10, IU/mL) and 199 (cells/µL), respectively. The demographic characteristics of the HIV-1-infected individuals are summarized in Table 1. Most of the 804 ART-experienced individuals (90.58%, 728/804) were treated with two NRTIs+PI/NNRTI during the whole antiviral process; 9.45% (76/804) of them had used INSTI-containing regimens (DTG (61.84%, 47/76), EVG (21.05%, 16/76), BIC (13.16%, 10/76) or RAL (3.95%, 3/76)).

**Figure 1:**
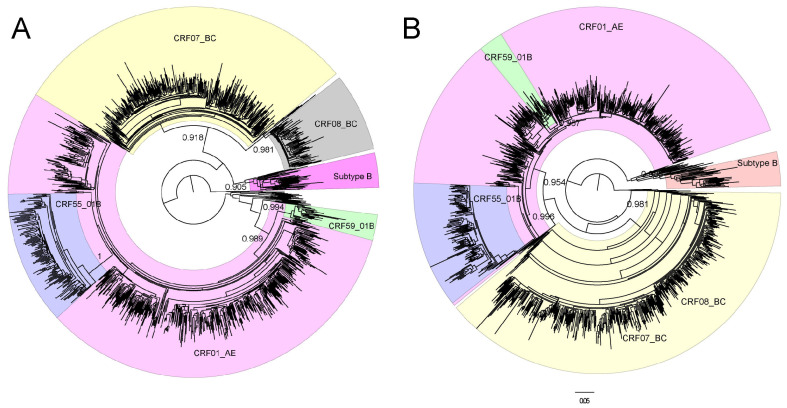
Maximum-likelihood phylogenetic tree based on PR/RT sequences (**A**) or IN sequences (**B**) obtained from 1104 individuals for which main genotypes were initially identified by COMET HIV. Reference sequences were downloaded from the Los Alamos HIV Sequence Database (https://www.hiv.lanl.gov/content/index, accessed on 7 September 2021); subtype H sequences were selected as the outgroup. The phylogenetic trees were generated using PhyML version 3.0 with GTR mode. aLTR values higher than 0.9 were used to identify lineages, as indicated at the corresponding nodes of the tree.

Complete IN sequences were obtained from all 1208 enrolled participants. There were no duplicate samples from the same subject at different time points. According to COMET HIV-1 analysis based on PR/RT sequences, CRF01_AE was the most frequently occurring genotype, with a proportion of 40.81% (493/1208), followed by CRF07_BC (28.64%, 346/1208) and CRF55_01B (28.64%, 346/1208). A total of six subtypes or circulating recombinant forms (CRFs) were confirmed (91.39%, 1104/1208) according to the ML phylogenetic tree based on 1104 PR/RT sequences (Figure 1A), which was constructed to determine the evolutionary relationship of these sequences. CRF07_BC and CRF08_BC cannot fall into clusters; meanwhile, sequences in CRF55_01B and CRF59_01B clusters cannot match the genotypes confirmed by the ML phylogenetic tree based on 1104 PR/RT sequences, as they lack the necessary breakpoints for subtyping (Figure 1B).

### 3.2. DRMs Associated with INSTIs in ART-Naive and ART-Experienced Participants

**Figure 2:**
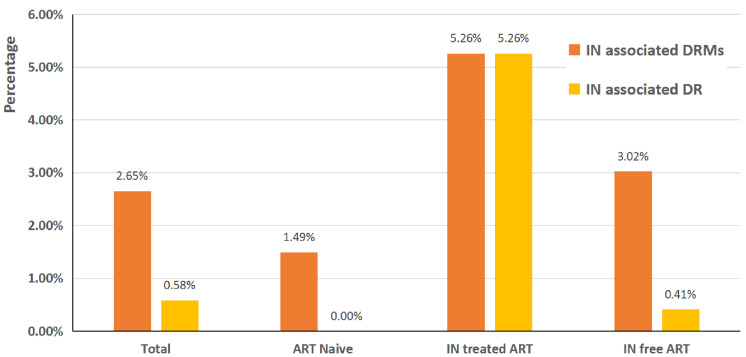
Percentages of INSTI-associated DRMs and DR among HIV-1-infected adults in Guangdong, China.

Of the 1208 individuals, 2.65% (32/1208) carried at least one INSTI major or accessory DRM. Among them, 1.49% (6/404) were obtained from ART-naive individuals, whereas 3.23% (26/804) were from ART-experienced individuals (Table 1, Figure 2).

A total of 38 INSTI-related DRMs were detected among the enrolled individuals. Approximately 2.32% (28/1208) harboured only INSTI accessory DRMs (ART experienced, 2.74% (22/804); ART naive, 1.49% (6/404)), 0.25% (3/1208) harboured only INSTI major DRMs (ART experienced, 0.37% (3/804)), and 0.08% (1/1208) harboured both INSTI major and accessory DRMs (ART experienced, 0.12% (1/804)).

**Figure 3:**
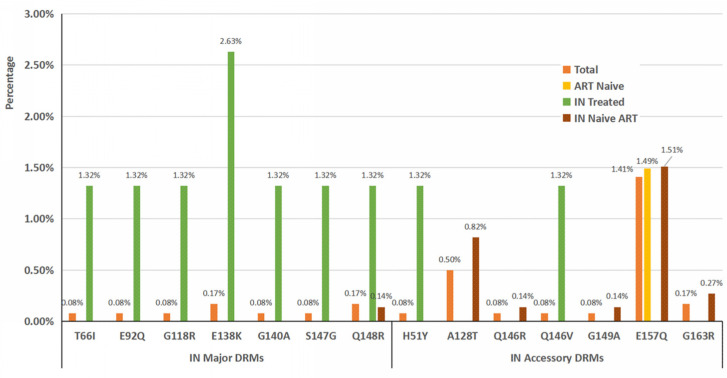
Percentages of INSTI-associated major and accessory DRMs among HIV-1-infected individuals in Guangdong, China.

E138K and Q148R (both 0.17%, 2/1208) were the most frequent mutations among INSTI major DRMs, and E157Q (1.41%, 17/1208) was the most frequent mutation among INSTI accessory DRMs. Moreover, INSTI major and accessory DRMs both varied among ART-naive and ART-experienced individuals (Figure 3).

### 3.3. DR Associated with INSTIs in ART-Naive and ART-Experienced Participants

**Figure 4:**
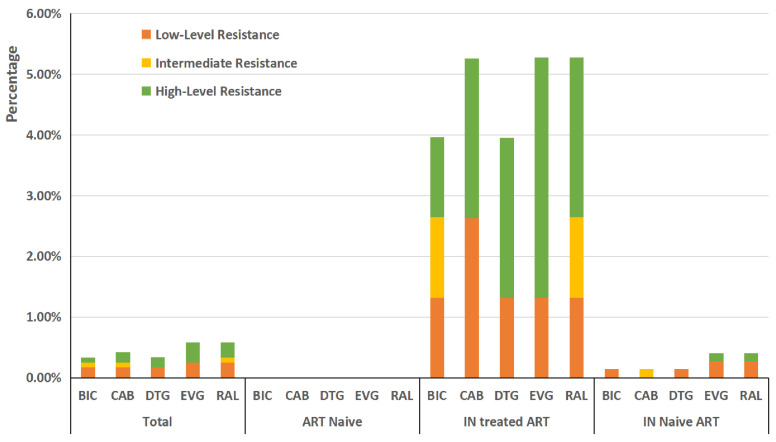
Percentages of resistance to INSTIs interpreted by Stanford HIV Drug Resistance Database in Guangdong.

According to the HIVdb program, 0.58% (7/1208) of the enrolled individuals carried INSTI-related DRMs associated with low-level, intermediate, or high-level resistance (Figure 4). The characteristics of the patients with INSTI-related mutations and the corresponding drug resistance levels are shown in Table 2. Four patients carrying INSTI-related major mutations all showed at least low-level resistance to INSTIs. Among the 28 patients carrying INSTI-related accessory mutations, we found only three cases carrying the H51Y or G163R mutation, which cause low-level resistance to INSTIs.

Seven patients with INSTI-related drug resistance were observed among the ART-experienced patients. Among them, four HIV-1 genotypes were identified (CRF01_AE, 1 CRF07_BC, 1 CRF55_01B and one unassigned genotype, respectively). Six individuals were male, and the dominant transmission route was HET (4/7), with a median age of 52 years (IQR: 29–73 years). Four individuals received INSTI-containing ART regimens (4/76, 5.26%), while the other three (3/728, 0.41%) were not recorded as utilizing INSTIs.

Conversely, no drug resistance to INSTIs was observed among treatment-naive patients. The prevalence of drug resistance to INSTIs among ART-experienced patients was 0.87% (7/804) (Figure 2). As shown in Figure 4, the percentages of resistance levels for the five INSTI drugs were all lower than 1.00%. Among the ART-experienced patients, the percentages of resistance were 0.37% (3/804), 0.50% (4/804), 0.37% (3/804), 0.50% (4/804), and 0.50% (4/804) for BIC, CAB, DTG, EVG, and RAL, respectively, and a greater proportion of high-level resistance to EVG was found (0.37%, 3/804) (Figure 4).

## 4. Discussion

INSTIs, a novel class of anti-HIV agents, show high activity in inhibiting HIV-1 replication and play a critical role in therapy for infection with this virus [18]. However, as shown in previous research, first-generation INSTIs (RAL and EVG) have lower genetic barriers and possible cross-resistance. While the second-generation INSTIs (DTG, CAB and BIC) have a higher resistance barrier [23], the surveillance of resistance to INSTIs is necessary with more frequent use of the single-tablet regimens (STRs) containing INSTIs.

HIV-1 has a high degree of natural variability due its lack of gene expression proofreading and high frequency of genetic recombination [24]. We observed six cases (1.49%) of the accessory INSTI resistance mutation E157Q among ART-naive individuals (Figure 3), which is a polymorphic mutation that appears to have little effect on INSTI susceptibility [25] and is common in ART-naive individuals with a very low proportion [21,26,27]. The absence of INSTI-associated drug resistance among ART-naive individuals suggests low circulation of INSTI-resistant variants prior to treatment in Guangdong, which is in accordance with reports both from other provinces and worldwide [21,24,28,29,30]. However, when compared with the prevalence of INSTI-related DRMs among ART-naive HIV-1-infected patients in 2018 (1.45%, 12/827) [21], no statistically significant difference in the prevalence of INSTI-related DRMs among ART-naive HIV-1 infections was observed in this study (χ^2^ = 0.002, *p* = 1.00 > 0.05). With the increasing number of patients using INSTI-containing regimens in Guangdong (patients who used INSTI-related regimens accounted for approximately 12.8% of all patients under ART in the Pearl River Delta region of Guangdong in 2021, unpublished data), surveillance should be reinforced with respect to the further choice of or transfer to INSTI-containing regimens.

Regarding ART-experienced individuals, most of the INSTI resistance mutations detected were accessory (71.88%, 23/32). The most frequent mutations were E157Q (1.37%, 11/804) and A128T (0.75%, 6/804), followed by G163R (0.25%, 2/804), H51Y (0.12%, 1/804), Q146R (0.12%, 1/804), Q146V (0.12%, 1/804), and G149A (0.12%, 1/804) (Figure 3). Except for G163R and H51Y, all these mutations are accessory and usually only reduce INSTI susceptibility when occurring in combination with other INSTI-resistance mutations [31]. G163R is nonpolymorphic in all subtypes except subtype F, and confers low-level resistance to EVG and RAL when appearing alone [32]. H51Y is a rare nonpolymorphic accessory mutation selected in patients receiving RAL or EVG, and minimally reduces EVG and possibly CAB susceptibility [33]. The patient carrying the H51Y mutation had used the BIC/FTC/TAF regimen as ART for four months, with HIV-1 viral load decreasing from 1.21 × 10^5^ IU/mL at baseline to 7.99 × 10^2^ IU/mL at DR test. The treatment failure in this case may have been due to the H51Y mutation, which needs to be confirmed by further phenotypic analysis.

For the ART-experienced individuals, 28.13% (9/32) of the INSTI resistance mutations detected are major resistance mutations. The most frequent major INSTI resistance mutations were E138K and Q148R (both 0.25%, 2/804), followed by T66I, E92Q, G118R, G140A, and S147G (all 0.12%, 1/804). All these major INSTI resistance mutations were detected in four patients (three of them had ever used regimens containing integrase), though the frequency of these major INSTI resistance mutations was very low (from 0.12% to 0.25%). E138K is a non-polymorphic mutation occurring in patients receiving RAL, EVG, and DTG [34]. In this study, two patients carrying the E138K mutation had used RAL and DTG. Q148R has been reported in patients with virological failure during DTG [35], which can reduce RAL and EVG susceptibility 30–100-fold [36]. Most of the major INSTI DRMs detected in this study may have been caused by the use of INSTIs, though the low rates of other INSTI DRMs (mainly accessory mutations) may have been due to natural polymorphisms in the IN region and the long-term use of 2NRTI + NNRTI regimens. Surveillance is necessary for the further use of INSTIs, especially in ART-experienced individuals.

According to previous studies [37,38], drug resistance to NNRTIs and/or NRTIs prior to treatment increases the risk of resistance to INSTIs. In this study, seven patients showed drug resistance to INSTIs, and six individuals were coupled with other class DRMs (Table 2). Whether other classes of DRM represent a risk factor for resistance to INSTIs remains to be investigated in the future.

According to previous studies [39,40,41,42,43], mutations outside of integrase gene, such as those in and near the 3′ polypurine tract (3′PPT) and in envelope glycoproteins, are able to confer resistance to INSTI. The continuous monitoring of mutations outside the IN gene warrants special importance in surveillance of development of drug resistance. As INSTIs are now widely used even in first-line therapy, we intend to focus on the continuous monitoring of the mutations outside the IN gene in subsequent research.

## 5. Conclusions

The overall prevalence of INSTI DR in Guangdong remains low (0.58%, 7/1208), which suggests that INSTIs currently have good applicability and that the use of INSTIs results in ideal viral suppression. Nevertheless, the proportion of DR in ART-experienced individuals was higher than that in ART-naive individuals (χ^2^ = 3.188, *p* = 0.088), and seven INSTI-related polymorphic major mutations were detected among HIV-1 patients in Guangdong, emphasizing the importance of monitoring drug resistance prior to administration of INSTI-containing regimens.

## Figures and Tables

**Table 1:** Demographic and Viral Characteristics of 1208 Study Population.

Characteristics	All Patient	ART Naive Patient	ART Experienced Patient
Patient, number	1208	404	804
Sex, number (%)			
Male	990 (81.95)	354 (87.62)	636 (79.10)
Female	218 (18.05)	50 (12.38)	168 (20.90)
Age, median (range)	43 (16–87)	33 (16–82)	91 (19–87)
HIV-1 RNA at DR test (Log10, IU/mL), number (%)	4.15 (2.32–7.51)	4.91 (2.88–7.51)	4.05 (2.32–6.66)
CD4 count at baseline (cells/µL), median(range)	199 (1–1302)	248 (1–1302)	162 (1–940)
HIV transmission route, number (%)			
MSM	326 (26.99)	188 (46.53)	138 (17.16)
Heterosexual	713 (59.02)	204 (50.5)	509 (63.31)
Intravenous drug users	89 (7.37)	7 (1.73)	82 (10.20)
SexIDU	5 (0.41)	1 (0.25)	4 (0.50)
Blood	3 (0.25)	0 (0.00)	3 (0.37)
Unknown	72 (5.96)	4 (0.99)	68 (8.46)
Geographical region, number (%)			
Pearl River Delta	660 (54.64)	289 (71.53)	371 (46.14)
Eastern	72 (5.96)	19 (4.70)	53 (6.59)
Western	308 (25.50)	36 (8.91)	272 (33.83)
Northern	168 (13.91)	60 (14.85)	108 (13.43)
Collection year, number (%)			
2021	616 (50.99)	66 (16.34)	550 (68.41)
2022	592 (49.01)	338 (83.66)	254 (31.59)
Confirm year, number (%)			
Before 2019	532 (44.04)	24 (5.94)	508 (63.18)
2019	106 (8.77)	3 (0.74)	103 (12.81)
2020	121 (10.02)	8 (1.98)	82 (10.20)
2021	189 (15.65)	109 (26.98)	111 (13.81)
2022	260 (21.52)	260 (64.36)	0 (0.00)
ART history (months), median (range)	-	-	41.02 (1–206)
HIV-1 genotypes based on PR/RT sequences, number (%)			
CRF01_AE	493 (40.81)	124 (30.69)	369 (45.90)
CRF07_BC	346 (28.64)	157 (38.86)	189 (23.51)
CRF08_BC	78 (6.46)	19 (4.70)	59 (7.34)
CRF55_01B	132 (10.93)	45 (11.14)	87 (10.82)
CRF59_01B	25 (2.07)	7 (1.73)	18 (2.24)
Subtype B	30 (2.48)	8 (1.98)	22 (2.74)
Other	104 (8.61)	44 (10.89)	60 (7.46)
IN mutation, number (%)	32 (2.65)	6 (1.49)	26 (3.23)
IN Drug Resistance, number (%)	7 (0.58)	0 (0.00)	7 (0.87)

**Table 2:** Characteristics of patients with IN-related mutations and the corresponding drug resistance level.

Sample ID	Group ^1^	Geographical Region	Sex	Age	Transmission Route	Genotype	IN DRM		IN DR ^2^					Ever Used Regimen Containing Integrase	Other Class DRMs
							Major	Accessory	BIC	CAB	DTG	EVG	RAL		
220111	Naive	PRD	Female	33	HET	08_BC	-	E157Q	S	S	S	P	P	No	No
220281	Naive	PRD	Female	49	HET	08_BC	-	E157Q	S	S	S	P	P	No	No
ZLQ08251	Naive	Northern	Female	37	HET	07_BC	-	E157Q	S	S	S	P	P	No	No
ZLQ08265	Naive	Northern	Male	23	MSM	01_AE	-	E157Q	S	S	S	P	P	No	No
ZLQ08266	Naive	Northern	Male	55	MSM	59_01B	-	E157Q	S	S	S	P	P	No	No
ZLQ08269	Naive	Northern	Male	51	HET	07_BC	-	E157Q	S	S	S	P	P	No	Yes
FX25834	ART	PRD	Male	58	HET	Other	E138K, G140A, S147G, Q148R	-	H	H	H	H	H	Yes/RAL	Yes
FX26041	ART	PRD	Male	39	HET	01_AE	-	H51Y	P	L	P	L	L	Yes/BIC	No
ZK01926	ART	PRD	Male	73	HET	01_AE	T66I, E92Q	-	L	L	L	H	M	Yes/EVG	Yes
ZK02467	ART	PRD	Male	32	MSM	01_AE	G118R, E138K	Q146V	M	H	H	H	H	Yes/DTG	Yes
FX25322	ART	PRD	Female	42	HET	07_BC	-	A128T	S	S	S	S	S	No	No
FX26691	ART	PRD	Male	39	MSM	01_AE	-	G149A	S	S	S	S	S	No	No
ZK01777	ART	Western	Male	59	Blood	01_AE	-	E157Q	S	S	S	P	P	No	No
ZK01784	ART	Western	Male	61	HET	07_BC	-	E157Q	S	S	S	P	P	No	Yes
ZK01822	ART	Western	Male	79	HET	Other	-	E157Q	S	S	S	P	P	No	Yes
ZK01839	ART	Western	Male	78	HET	Other	-	E157Q	S	S	S	P	P	No	Yes
ZK01936	ART	PRD	Female	41	HET	07_BC	-	A128T	S	S	S	S	S	No	No
ZK02078	ART	Western	Male	35	IDU	07_BC	-	A128T	S	S	S	S	S	No	No
ZK02106	ART	Eastern	Male	29	MSM	55_01B	Q148R	-	L	M	L	H	H	No	Yes
ZK02277	ART	Northern	Male	73	HET	07_BC	-	E157Q	S	S	S	P	P	No	Yes
ZK02406	ART	Western	Female	71	HET	01_AE	-	G163R	S	S	S	L	L	No	Yes
ZK02501	ART	Eastern	Female	27	HET	01_AE	-	E157Q	S	S	S	P	P	No	No
ZK02503	ART	Eastern	Male	46	HET	55_01B	-	E157Q	S	S	S	P	P	No	Yes
ZK02513	ART	Western	Male	71	HET	07_BC	-	A128T	S	S	S	S	S	No	Yes
ZK02543	ART	Western	Male	52	IDU	07_BC	-	G163R	S	S	S	L	L	No	Yes
ZK02548	ART	Western	Male	36	IDU	07_BC	-	E157Q	S	S	S	P	P	No	Yes
ZK02590	ART	Northern	Male	45	IDU	07_BC	-	E157Q	S	S	S	P	P	No	No
ZK02723	ART	PRD	Male	40	HET	08_BC	-	E157Q	S	S	S	P	P	No	Yes
ZK02810	ART	PRD	Male	54	IDU	07_BC	-	A128T	S	S	S	S	S	No	Yes
ZK02853	ART	PRD	Male	46	IDU	07_BC	-	A128T	S	S	S	S	S	No	No
ZK02870	ART	Western	Male	70	HET	08_BC	-	Q146R	S	S	S	S	S	No	Yes
ZK02877	ART	Western	Male	52	HET	07_BC	-	E157Q	S	S	S	P	P	No	No

^1^ Naive: ART naive; ART: ART Experienced. ^2^ S: Susceptible; P: Potential Low-Level Resistance; L: Low-Level Resistance; M: Intermediate Resistance; H: High-Level Resistance.

## Data Availability

The data that support the findings of this study are available from the corresponding author upon reasonable request.

## Abbreviations

3′PPT: 3′ polypurine tract; aLRT: approximate likelihood ratio test; ADR: Acquired drug resistance; BIC: bictegravir; CAB: cabotegravir; cART: Combined antiretroviral therapy; CRFs: circulating recombinant forms; DR: drug resistance; DRM: drug resistance mutation; DTG: dolutegravir; EVG: elvitegravir; HET: Heterosexual; IDU: intravenous drug users; IN: Integrase; INSTI: Integrase strand-transfer inhibitor; ML: maximum likelihood; MSM: men who have sex with men; NNRTIs: nonnucleoside reverse transcriptase inhibitors; NRTIs: nucleoside reverse transcriptase inhibitors; PIs: protease inhibitors; PLWHIV: people living with HIV and AIDS; RAL: raltegravir; RT: reverse transcriptase; STRs: single-tablet regimens; TDR: transmitted drug resistance; WHO: World Health Organization.

## References

[1] De Cock K.M., Jaffe H.W., Curran J.W. (2021). Reflections on 40 Years of AIDS. Emerg. Infect. Dis..

[2] Delpech V. (2022). The HIV epidemic: Global and United Kingdom trends. Medicine.

[3] WHO Latest HIV Estimates and Updates on HIV Policies Uptake. December 2021. https://cdn.who.int/media/docs/default-source/hq-hiv-hepatitis-and-stis-library/2021_global_summary_web_v32.pdf?sfvrsn=4b8815ad_37.

[4] National Center for AIDS & STD Control and Prevention, The Chinese Center for Disease Control and Prevention (CDC) (2021). Annals of Information on Comprehensive Prevention and Treatment for AIDS, STD and Hepatitis C.

[5] Guangdong Provincial Center for Disease Control and Prevention Thirty Five Years Warning Record of Anti-HIV/AIDS in Guangdong Provience China. https://www.cn-healthcare.com/articlewm/20211207/content-1292810.html.

[6] Nosyk B., Audoin B., Beyrer C., Cahn P., Granich R., Havlir D., Katabira E., Lange J., Lima V.D., Patterson T. (2013). Examining the evidence on the causal effect of HAART on transmission of HIV using the Bradford Hill criteria. AIDS.

[7] Lima V.D., Brumme Z.L., Brumme C., Sereda P., Krajden M., Wong J., Guillemi S.A., Henry B., Hogg R.S., STOP HIV/AIDS Study Group (2020). The Impact of Treatment as Prevention on the HIV Epidemic in British Columbia, Canada. Curr. HIV/AIDS Rep..

[8] Crowell T.A., Danboise B., Parikh A., Esber A., Dear N., Coakley P., Kasembeli A., Maswai J., Khamadi S., Bahemana E. (2020). Pretreatment and Acquired Antiretroviral Drug Resistance Among Persons Living with HIV in Four African Countries. Clin. Infect. Dis..

[9] Girón-Callejas A., García-Morales C., Mendizabal-Burastero R., Román M., Tapia-Trejo D., Pérez-García M., Quiroz-Morales V.S., Juárez I.S., Ravasi G., Vargas C. (2019). High levels of pretreatment and acquired HIV drug resistance in Nicaragua: Results from the first nationally representative survey, 2016. J. Int. AIDS Soc..

[10] Arimide D.A., Amogne M.D., Kebede Y., Balcha T.T., Adugna F., Ramos A., DeVos J., Zeh C., Agardh A., Chang J.C.-W. (2022). High Level of HIV Drug Resistance and Virologic Nonsuppression Among Female Sex Workers in Ethiopia: A Nationwide Cross-Sectional Study. JAIDS J. Acquir. Immune Defic. Syndr..

[11] Bissio E., Barbás M.G., Kademián S., Bouzas M.B., Salomón H., Cudolá A., Giuliano S.F., Falistocco C. (2017). Prevalence of Rilpivirine Resistance in People Starting Antiretroviral Treatment in Argentina. Antivir. Ther..

[12] Jordan M.R., Penazzato M., Cournil A., Vubil A., Jani I., Hunt G., Carmona S., Maphalala G., Mthethwa N., Watera C. (2017). Human Immunodeficiency Virus (HIV) Drug Resistance in African Infants and Young Children Newly Diagnosed With HIV: A Multicountry Analysis. Clin. Infect. Dis..

[13] Buzón M.J., Marfil S., Puertas M.C., Garcia E., Clotet B., Ruiz L., Blanco J., Martinez-Picado J., Cabrera C. (2008). Raltegravir susceptibility and fitness progression of HIV type-1 integrase in patients on long-term antiretroviral therapy. Antivir. Ther..

[14] Podany A.T., Scarsi K.K., Fletcher C.V. (2017). Comparative Clinical Pharmacokinetics and Pharmacodynamics of HIV-1 Integrase Strand Transfer Inhibitors. Clin. Pharmacokinet..

[15] Wang Y., Gu S.-X., He Q., Fan R. (2021). Advances in the development of HIV integrase strand transfer inhibitors. Eur. J. Med. Chem..

[16] Department of Health and Human Services (DHHS) Panel on Antiretroviral Guidelines for Adults and Adolescents (2016). Guidelines for the Use of Antiretroviral Agents in HIV-1-Infected Adults and Adolescents. Department of Health and Human Services. http://aidsinfo.nih.gov/ContentFiles/AdultandAdolescentGL.pdf.

[17] AIDS and Hepatitis C Professional Group, Society of Infectious Diseases, Chinese Medical Association, Chinese Center for Disease Control and Prevention (2018). Chinese guidelines for diagnosis and treatment of HIV/AIDS (Version 2018). Zhonghua Nei Ke Za Zhi.

[18] Smith S.J., Zhao X.Z., Passos D.O., Lyumkis D., Burke T.R., Hughes S.H. (2021). Integrase Strand Transfer Inhibitors Are Effective Anti-HIV Drugs. Viruses.

[19] Zolopa A., Sax P.E., DeJesus E., Mills A., Cohen C., Wohl D., Gallant J.E., Liu H.C., Plummer A., White K.L. (2013). A Randomized Double-Blind Comparison of Coformulated Elvitegravir/Cobicistat/Emtricitabine/Tenofovir Disoproxil Fumarate Versus Efavirenz/Emtricitabine/Tenofovir Disoproxil Fumarate for Initial Treatment of HIV-1 Infection. JAIDS J. Acquir. Immune Defic. Syndr..

[20] Jiang J., Xu X., Guo W., Su J., Huang J., Liang B., Chen H., Zang N., Liao Y., Ye L. (2016). Dolutegravir (DTG, S/GSK1349572) combined with other ARTs is superior to RAL- or EFV-based regimens for treatment of HIV-1 infection: A meta-analysis of randomized controlled trials. AIDS Res. Ther..

[21] Lan Y., Li L., Chen W., Deng X., Li J., Fan Q., Cai X., Cai W., Hu F. (2020). Absence of Integrase Inhibitor-Associated Resistance Among Antiretroviral Therapy-Naïve HIV-1-Infected Adults in Guangdong Province, China, in 2018. Infect. Drug Resist..

[22] Guindon S., Dufayard J.-F., Lefort V., Anisimova M., Hordijk W., Gascuel O. (2010). New Algorithms and Methods to Estimate Maximum-Likelihood Phylogenies: Assessing the Performance of PhyML 3.0. Syst. Biol..

[23] Anstett K., Brenner B., Mesplede T., Wainberg M.A. (2017). HIV drug resistance against strand transfer integrase inhibitors. Retrovirology.

[24] Neher R.A., Leitner T. (2010). Recombination Rate and Selection Strength in HIV Intra-patient Evolution. PLoS Comput. Biol..

[25] Bradley-Stewart A., Urcia C., MacLean A., Aitken C., Gunson R. (2017). HIV-1 integrase inhibitor resistance among treatment naïve patients in the West of Scotland. J. Clin. Virol..

[26] Masoud S., Kamori D., Barabona G., Mahiti M., Sunguya B., Lyamuya E.F., Ueno T. (2020). Circulating HIV-1 Integrase Genotypes in Tanzania: Implication on the Introduction of Integrase Inhibitors-Based Antiretroviral Therapy Regimen. AIDS Res. Hum. Retrovir..

[27] López P., Tirado G., Arias A., Sánchez R., Rodríguez-López E., Rivera-Amill V. (2021). Short Communication: Integrase Strand Transfer Inhibitors Drug Resistance Mutations in Puerto Rico HIV-Positive Individuals. Int. J. Environ. Res. Public Health.

[28] Liu L., Dai L., Yao J., Pan P., Li L., Liu Z., An X., Sun L., Wu H., Su B. (2019). Lack of HIV-1 integrase inhibitor resistance among 392 antiretroviral-naïve individuals in a tertiary care hospital in Beijing, China. AIDS.

[29] Yu F., Li Q., Wang L., Zhao H., Wu H., Yang S., Tang Y., Xiao J., Zhang F. (2022). Drug Resistance to HIV-1 Integrase Inhibitors Among Treatment-Naive Patients in Beijing, China. Pharm. Pers. Med..

[30] Yang Z., Yang X., Deng X., Wei S., Liu J., Ma J., Zhao Q., Huo Y. (2021). Prevalence of integrase strand transfer inhibitor (INSTIs) resistance mutations in Henan Province, China (2018–2020). Infection.

[31] Feng L., Sharma A., Slaughter A., Jena N., Koh Y., Shkriabai N., Larue R., Patel P.A., Mitsuya H., Kessl J.J. (2013). The A128T Resistance Mutation Reveals Aberrant Protein Multimerization as the Primary Mechanism of Action of Allosteric HIV-1 Integrase Inhibitors. J. Biol. Chem..

[32] Rhee S.-Y., Sankaran K., Varghese V., Winters M.A., Hurt C.B., Eron J.J., Parkin N., Holmes S.P., Holodniy M., Shafer R.W. (2016). HIV-1 Protease, Reverse Transcriptase, and Integrase Variation. J. Virol..

[33] Quashie P.K., Oliviera M., Veres T., Osman N., Han Y.-S., Hassounah S., Lie Y., Huang W., Mesplède T., Wainberg M.A. (2015). Differential Effects of the G118R, H51Y, and E138K Resistance Substitutions in Different Subtypes of HIV Integrase. J. Virol..

[34] Naeger L.K., Harrington P., Komatsu T., Deming D. (2016). Effect of Dolutegravir Functional Monotherapy on HIV-1 Virological Response in Integrase Strand Transfer Inhibitor Resistant Patients. Antivir. Ther..

[35] Blanco J.L., Rojas J., Paredes R., Negredo E., Mallolas J., Casadella M., Clotet B., Gatell J.M., De Lazzari E., Martínez E. (2018). Dolutegravir-based maintenance monotherapy versus dual therapy with lamivudine: A planned 24 week analysis of the DOLAM randomized clinical trial. J. Antimicrob. Chemother..

[36] Abram M.E., Hluhanich R.M., Goodman D.D., Andreatta K.N., Margot N.A., Ye L., Niedziela-Majka A., Barnes T.L., Novikov N., Chen X. (2013). Impact of Primary Elvitegravir Resistance-Associated Mutations in HIV-1 Integrase on Drug Susceptibility and Viral Replication Fitness. Antimicrob. Agents Chemother..

[37] Siedner M.J., Moorhouse M.A., Simmons B., de Oliveira T., Lessells R., Giandhari J., Kemp S.A., Chimukangara B., Akpomiemie G., Serenata C.M. (2020). Reduced efficacy of HIV-1 integrase inhibitors in patients with drug resistance mutations in reverse transcriptase. Nat. Commun..

[38] Ndashimye E., Arts E.J. (2021). Dolutegravir response in antiretroviral therapy naïve and experienced patients with M184V/I: Impact in low-and middle-income settings. Int. J. Infect. Dis..

[39] Malet I., Delelis O., Nguyen T., Leducq V., Abdi B., Morand-Joubert L., Calvez V., Marcelin A.-G. (2019). Variability of the HIV-1 3’ polypurine tract (3’PPT) region and implication in integrase inhibitor resistance. J. Antimicrob. Chemother..

[40] Dekker J.G., Klaver B., Berkhout B., Das A.T. (2022). Mutations in the HIV-1 3′-Polypurine Tract Can Confer Dolutegravir Resistance. Antimicrob. Agents Chemother..

[41] Wei Y., Sluis-Cremer N. (2021). Mutations in the HIV-1 3′-Polypurine Tract and Integrase Strand Transfer Inhibitor Resistance. Antimicrob. Agents Chemother..

[42] Malet I., Subra F., Charpentier C., Collin G., Descamps D., Calvez V., Marcelin A.-G., Delelis O. (2017). Mutations Located outside the Integrase Gene Can Confer Resistance to HIV-1 Integrase Strand Transfer Inhibitors. mBio.

[43] Hachiya A., Kubota M., Shigemi U., Ode H., Yokomaku Y., Kirby A.K., Sarafianos S.G., Iwatani Y. (2022). Specific mutations in the HIV-1 G-tract of the 3′-polypurine tract cause resistance to integrase strand transfer inhibitors. J. Antimicrob. Chemother..

